# Augmenting art crossmodally: possibilities and pitfalls

**DOI:** 10.3389/fpsyg.2025.1605110

**Published:** 2025-07-14

**Authors:** Charles Spence, Nicola Di Stefano

**Affiliations:** ^1^Crossmodal Research Laboratory, Department of Experimental Psychology, Oxford University, Oxford, United Kingdom; ^2^Institute of Cognitive Sciences and Technologies, National Research Council, Rome, Italy

**Keywords:** art, audiovisual, crossmodal, Gestalt, intersensoriality, multisensory, sensory translation, sensory augmentation

## Abstract

In this narrative historical review, we take a closer look at the question of whether it is possible to augment works of art through crossmodal (specifically audiovisual) means. We start by highlighting an important distinction between three classes of audiovisual crossmodal correspondence: Namely those operating on individual sensory stimuli (so-called basic correspondences), those operating on dynamically-changing stimuli, or else on combinations of unisensory stimuli (so-called mid-level correspondences), and those operating on complex and often aesthetically-meaningful stimuli, such as music and paintings. We also highlight another important distinction between the literature on crossmodal matching and that dedicated to demonstrating crossmodal effects. The latter distinction aligns, in some sense, onto the distinction between crossmodal mapping and crossmodal effects. Although it may not be possible, in any meaningful sense, to translate works of art from one modality into another, that does not deny the possibility of augmenting a work of art by the deliberate addition of stimulation presented to another sensory modality. The aims and objectives of those who have attempted to augment works of art by introducing additional sensory stimulation are discussed. We also draw attention to a number of challenges and/or pitfalls (such as the distraction offered by recourse to the phenomenon of synaesthesia) for those interested in augmenting auditory/visual art crossmodally.

## 1 Introduction

In recent decades, there has been a growing awareness within the cognitive neurosciences that the senses do not operate in isolation (Barlow and Mollon, 1982), and that crossmodal interactions are the norm, not the exception (see Calvert et al., 2004).^1^ Crossmodality refers to the way in which the stimuli presented in one sense may influence people's perception of the stimuli that happen to be presented in another sense. Crossmodality is different from the term multisensory, with the latter referring to those situations when the senses come together to deliver a multisensory representation of an object or event (Stein et al., 2010). Classic examples of multisensory perception include everything from audiovisual speech perception to multisensory flavor perception. Crossmodal effects, by contrast, are more often observed when seemingly unrelated unisensory stimuli are presented at around the same time, such as when the presentation of a bright light makes a simultaneously-presented sound appear louder than it otherwise would (see Spence, 2018, for a review).

One of the most exciting recent areas in crossmodal research concerns the crossmodal correspondences. This is the name given to the almost synaesthesia-like surprising connections between features, attributes, or dimensions of experience presented in different senses. Often confused with synaesthesia (in that both phenomena are surprising when first you hear about them), they are fundamentally different (see Deroy and Spence, 2013). For instance, there is typically a crossmodal association but no inducer-concurrent mapping in the case of crossmodal correspondences. Furthermore, crossmodal correspondences tend to be consensually shared across groups of people, whereas the inducer-concurrent mapping in the context of synaesthesia is, by definition, idiosyncratic between one synaesthete and the next. It is the crossmodal correspondences that we are interested.

In this narrative historical review, we first introduce an important distinction between three classes of audiovisual crossmodal correspondence: Namely those operating on individual sensory stimuli (so-called basic correspondences), those operating on dynamically-changing stimuli, or else on combinations of unisensory stimuli (so-called mid-level correspondences), and those operating on complex and often aesthetically-meaningful stimuli, such as music and paintings (Section 1.1). We also highlight an important distinction between the literature on crossmodal matching and that dedicated to demonstrating crossmodal effects. The latter distinction aligns, in some sense, with the distinction between crossmodal mapping and crossmodal effects. In Section 1.2, we briefly consider the kinds of explanations that have been put forward to account for crossmodal correspondences to date. Thereafter, in Sections 2–4, we review the evidence concerning audiovisual crossmodal matching and crossmodal effects operating at the level of basic, mid-level, and complex crossmodal correspondences, respectively.

In Section 5, we build on this groundwork by reflecting on the putative existence of crossmodal art. We take a closer look at the question of whether it is possible to augment works of art through crossmodal (specifically audiovisual) means. We show how the phenomenon of synaesthesia, at least as conceptualized by cognitive neuroscientists fails to enlighten theorizing when it comes to thinking about the social/structural construction of meaning in the context of the crossmodal augmentation of art (Dimova, 2024; Lévi-Strauss, 1997; Spence and Deroy, 2013). We discuss how although it may not be possible, in any meaningful sense, to translate works of art from one modality into another (see Spence and Di Stefano, 2024a), that does not deny the possibility of augmenting a work of art by the deliberate addition of stimulation presented to another sensory modality. Finally, we discuss the aims and objectives of those who have attempted to augment works of art by introducing additional sensory stimulation. The intriguing notion of intersensoriality is also outlined. This term has been introduced by Howes (2025) to refer to art that crosses sensory borders (Classen, 1998). We draw attention to a number of the challenges and/or pitfalls for those interested in wanting to augment auditory/visual art crossmodally (Section 6). We conclude, in Section 7, by challenging the notion, implicit in much creative practice, that elemental correspondences (that is, crossmodal correspondences based on simple stimulus properties), provide the most relevant insights when it comes to augmenting art crossmodally, given that works of art are mostly complex, multi-elemental, and rich in semantic and emotional meaning.

### 1.1 Basic, mid-level, and complex crossmodal correspondences: a novel typology

Audiovisual crossmodal correspondences can be grouped into different classes based on the (perceived) complexity of the stimuli involved. In particular, while some researchers have chosen to study crossmodal correspondences between basic (i.e., individual) sensory stimuli (i.e., specific features or dimensions), others have chosen to focus on crossmodal correspondences (and Gestalt grouping) in the case of what might be called mid-level correspondences instead (i.e., those correspondences that are experienced between combinations of unisensory stimuli, such as an auditory melody and a sequence of visual stimuli).^2^ A third group of studies, meanwhile, has focused on crossmodal correspondences involving more complex and often semantically-meaningful stimuli, such as, for example, works of art (primarily paintings and classical music) and other popular entertainment media (such as film clips, TV adverts, and popular music selections). While the majority of the research that has been published to date has tended to focus on those correspondences that can be demonstrated between stimuli at the same level of complexity, a number of studies have also investigated crossmodal correspondences operating across levels (as when color properties are associated with pieces of music).

Basic audiovisual crossmodal correspondences operate between discrete unimodal sensory stimuli, such as, for example, pure tones, color patches, and sounds and lights varying in terms of their intensity (i.e., loudness and brightness). It has sometimes been suggested that certain of these basic crossmodal correspondences may reflect the existence of amodal sensory dimensions, such as intensity, shape, texture, brightness, etc. (e.g., Bond and Stevens, 1969; Ellermeier et al., 2021; Lewkowicz and Turkewitz, 1980; Marks et al., 1987; Root and Ross, 1965). However, that possibility has been rejected recently, at least in the case of sensory correspondences (see Spence and Di Stefano, 2024b, for a review). Rather, at least according to Spence and Di Stefano, putatively amodal dimensions of experience, such as for sensory intensity, should be reconceptualized in terms of a crossmodal correspondence instead (e.g., between auditory intensity and visually-perceived brightness, say). Similarly, Spence's (2015) suggestion from a decade ago that the crossmodal correspondences between individual sensory attributes might operate on the basis of perceived similarity has also recently been discounted, at least in the case of audition and vision (Di Stefano and Spence, 2024a).^3^

Mid-level correspondences typically operate between structured combinations of unisensory stimuli, such as short sequences of sounds (that may make up a melody or a spatiotemporally arranged pattern of visual stimuli). Numerous studies, often in the Gestalt tradition, have demonstrated how the grouping of sequences of stimuli presented in one sensory modality may influence the presentation of stimuli presented in the other modality, as for example, in the context of the so-called “crossmodal dynamic capture effect” (Soto-Faraco et al., 2003; see Spence et al., 2007, for a review). Other research, meanwhile, has demonstrated the existence of crossmodal effects in a range of other experimental situations (O'Leary and Rhodes, 1984; see also Huang et al., 2012; Rahne et al., 2008; Schwartz et al., 2012). So, for example, crossmodal effects of auditory perceptual grouping on visual perceptual grouping, and *vice versa*, have been reported. At the same time, however, a number of studies have also demonstrated mid-level crossmodal correspondences that are seemingly more metaphorical or analogical in nature (e.g., Ravignani and Sonnweber, 2017; Wagner et al., 1981; though see also Jewanski, 2010; Lindholm, 2022). A separate literature has demonstrated how temporal synchrony, and the presentation of correlated inputs to different senses, can also facilitate crossmodal binding (e.g., Lin et al., 2022; Parise et al., 2013, 2012).

In parallel, various researchers have explored audiovisual correspondences using more complex and semantically-rich stimuli. In such cases, the complexity of the stimuli involved means that it is harder to explain any crossmodal correspondences that are observed based on specific individual auditory/visual physical stimulus attributes/dimensions (e.g., such as frequency and hue; see Duthie, 2013; Duthie and Duthie, 2015), thus leading researchers to suggest the existence of features that may be capable of mediating those associations at more of a cognitive level. So, for instance, Albertazzi et al. (2015) demonstrated the existence of consistent audiovisual associations between highly-complex stimuli consisting of a series of 15 paintings by an Italian artist and 15 music excerpts from the classic guitar repertoire (e.g., Villas Lobos, Albeniz). These associations were explained using the semantic differential technique based on perceptual and emotional associations (e.g., bright and calm, respectively) (see also Cowles, 1935, for an early study, Spence, 2020a, for a review, and Iosifyan et al., 2022, for a more recent study documenting crossmodal associations between paintings and sounds). According to the “emotional mediation hypothesis”, those stimuli that are presented in different sensory domains are more likely to be matched if they share similar affective meanings (see Spence, 2020a, for a review). This has emerged as one of the most powerful accounts of this class of complex crossmodal correspondences, and is especially powerful in helping to explain audiovisual associations (e.g., Di Stefano et al., 2024, for cross-cultural evidence), though the approach has also been applied to help explain crossmodal associations involving olfactory stimuli (e.g., Di Stefano et al., 2022; Schifferstein and Tanudjaja, 2004; Spence, 2020b) and even taste stimuli as well (Spence and Levitan, 2021).

At the same time, however, a separate distinction can be made between those studies that have merely sought to establish the existence of crossmodal correspondences between the stimuli presented at a particular level (i.e., basic, mid-level, or complex)—crossmodal matching studies—and those studies that have looked to determine whether there are any crossmodal effects, evidenced by the presence of stimuli in one modality having an impact on those presented in the other modality (see Table 1). The general belief amongst researchers would appear to be that there should be an increased probability of crossmodal binding or multisensory integration (occasionally resulting in the perceived unification of the component sensory stimuli; Eisenstein, 1947; Wells, 1980) when stimuli are crossmodally congruent as compared to when presented with crossmodally incongruent pairings instead (Iwamiya, 2013).

**Table 1:** Broad categorization of different types of crossmodal correspondence.

**Type of correspondence**	**Stimuli involved**	**Crossmodal matching (CM)**	**Crossmodal effect (CE) or multisensory interaction**
Basic (or low-level) correspondence (more sensory than emotional)	Pure tone, loudness, pitch; colour patch, hue, brightness.	Multiple crossmodal correspondences documented, though disagreement about the pitch-hue mapping.	Coupling priors, unity effect (Parise and Spence, 2009)
Mid-level correspondence	Rhythm, musical mode; colour harmony, spatiotemporal patterns	Metaphoric matching, structural similarity; numerosity correspondence, analogical mapping.	Gestalt grouping (O'Leary and Rhodes, 1984); Modulation of crossmodal dynamic capture effect (Spence, 2015; Spence et al., 2007)
Complex (or high- level) more emotional than sensory; Stylistic correspondences (Hasenfus et al., 1983; Siefkes and Arielli, 2015)	Painting, piece of music, film clips, TV adverts; ballet/dance (Opera excluded as it is intrinsically audiovisual)	Music matched to painting; Painting chosen to match music. Visuals matched to music; movements/body shapes or gesture and music? Light show during concert?	Does music the change viewer's interpretation of painting or film? What is the impact of presenting incongruent music? Occasional reports of unification of inputs (e.g., in Scriabin's Prometheus, Wells, 1980; Video jockeys matching visuals to music; e.g., Jean-Michel Jarré)

While crossmodal correspondences operate between sensory stimuli presented at different levels of complexity (e.g., between individual auditory stimuli and complex visual stimuli), the majority of research that has been published to date has tended to present stimuli at the same level of complexity in both the auditory and visual modalities. Nevertheless, a few studies have been published documenting crossmodal correspondences between stimuli at different levels, such as, for example, demonstrating crossmodal correspondences between short snippets of music and isolated color patches (e.g., Palmer et al., 2013; Whiteford et al., 2018).

There are two possibly controversial claims (or observations) that we wish to make in this review: The first is that the mere existence of a crossmodal correspondence, as revealed by research on crossmodal matching, has no necessary implications for the existence of a crossmodal effect; The second is that the existence of crossmodal correspondences operating between individual sensory stimuli has no necessary implications as far as the existence of crossmodal effects operating between complex stimuli are concerned. That is, many crossmodal effects have been demonstrated in the literature without any consideration of the crossmodal congruency, or otherwise, of the component unimodal stimuli (see London, 1954; Welch and Warren, 1980, 1986, for reviews). So, for example, the presentation of a tone has been reported to enhance the rated brightness of a simultaneously-presented light (Stein et al., 1996; though see Odgaard et al., 2003), or vice versa (Odgaard et al., 2004). In this case, there was absolutely no consideration of whether the frequency of the sound was in any sense congruent (or incongruent) with the color of the light. Meanwhile, many studies have demonstrated how crossmodal effects (albeit different ones) occur in the case of both congruent and incongruent combinations of sensory stimuli (Chen et al., 2025). So, for example, Chen and colleagues reported that auditory stimuli influenced people's aesthetic perception of pictures regardless of the semantic or spatial congruency of the component stimuli.

It sometimes appears to be implicitly assumed by researchers working in the area (i.e., in crossmodal correspondences research) that the existence of a crossmodal effect can be taken as evidence of the existence of a crossmodal correspondence between one or several of the sensory features or dimensions involved. As such, the view that we wish to advocate here is that the existence of a crossmodal correspondence can only be convincingly demonstrated by the results of crossmodal matching studies (that is, in studies where the participants are explicitly asked to pick the best match of the available options that have been provided to them^4^ or, in more refined/informative protocols, to rate the extent to which all of the presented stimuli from different sensory domains match one another). While it would seem likely that crossmodal correspondences, as demonstrated in so many crossmodal matching studies, will likely give rise to measurable differences when a participant's responses to congruent vs. incongruent combinations of crossmodal stimuli are compared (Parise and Spence, 2009, 2012), it is important to remember that many (perhaps even the majority of) crossmodal effects may actually have little to do with crossmodal correspondences.

### 1.2 Explaining crossmodal correspondences

Over the years, a number of different mechanisms have been put forward to try to explain crossmodal correspondences (Motoki et al., 2023; Spence, 2011; Spence and Di Stefano, 2024a). These include statistical, structural (perhaps better referred to as physiological correspondences; see Spence and Di Stefano, 2024a), semantic (though again possibly better referred to as “lexical”, see Walker, 2012) as well as the emotional account (Spence, 2020a) (see Table 2). It is important to recognize that the most appropriate explanation for a given crossmodal correspondence is to some extent independent of the complexity of the component unisensory stimuli that are involved. That said, claims regarding the existence of amodal sensory and similarity-based crossmodal correspondences have typically been made in relation to individual sensory stimuli. Meanwhile, Gestalt grouping, as well as spatiotemporal synchrony, is more often mentioned as the most probable explanation for any crossmodal effects operating at the level of mid-level correspondences (Spence, 2015). Emotional mediation tends to be the most frequently mentioned explanation of audiovisual correspondences between complex and aesthetically-meaningful stimuli, such as, for example, music and painting/images (e.g., Di Stefano et al., 2024). That said, emotional mediation is occasionally raised as a possible explanation in the case of crossmodal correspondences between individual perceptual stimuli, while basic sensory correspondences (e.g., between pitch and size) could potentially also contribute to what might, at first, be taken as stylistic correspondences (see Duthie, 2013; Duthie and Duthie, 2015; Siefkes and Arielli, 2015).

**Table 2:** Summary of the various different types of crossmodal correspondence that have been proposed that have to connect auditory and visual stimuli and selected literature sources suggested and/or supported them.

**Type of correspondence**	**Description/explanation**
Structural	Correspondence based on structural alignment of stimulus dimensions (e.g., both pitch and hue being circular dimensions)
Physical	Correspondence based on fact that stimuli themselves are of a similar type (e.g., both sound and light being considered as waves)
Physiological^*^	Correspondence based on similar neural hypothetical similar neural encoding principle (e.g., increases in intensity being represented by increased neural firing)
Psychological -perceptual (-Amodal)	Correspondence based on perceptual similarity between the component stimuli (sometimes referred to as intersensory equivalence)
Mood-based/affective/emotional-mediation	Correspondence based on mood/emotion associated with each of the component stimuli being similar
Associative/statistical	Correspondence based on associative learning/internalization of crossmodal statistical regularities in environment

^*^Note that Spence (2011) originally labeled this category ‘Structural'. However, given that this label had already been introduced 20 years earlier by Sebba (1991) to describe dimensional alignment of perceptual space, it would seem more appropriate to call this category “Physiological”, given that that is the putative cause/source of the correspondence.

## 2 Crossmodal correspondences between simple sensory stimuli

One of the most extensively studied areas in research on audiovisual correspondences concerns the associations that have been demonstrated between individual sensory stimuli (or attributes), such as pitch, brightness, or loudness. Over the last 50 years or so, a very wide array of crossmodal matching studies has been published. More recently, a number of studies demonstrating crossmodal effects on perceptual binding as well as in a variety of speeded response tasks that are modulated by the crossmodal correspondences that exist between individual sensory stimuli (attributes or sensory dimensions) have also appeared in the literature; it is to this research that we turn next.

### 2.1 Crossmodal matching

Many published studies have documented crossmodal correspondences between simple stimuli, or stimulus dimensions (see Spence, 2011; Spence and Sathian, 2020, for reviews of the literature on basic audiovisual correspondences). In fact, the literature reveals a multitude of crossmodal correspondences between, for instance, auditory pitch and a variety of visually-presented dimensions: These include size (e.g., Anikin and Johansson, 2019; Bonetti and Costa, 2018; Evans and Treisman, 2010; Gallace and Spence, 2006; Mondloch and Maurer, 2004), shape/angularity (Marks, 1987; Murari et al., 2020; Parise and Spence, 2012), lightness, brightness (e.g., Brunel et al., 2015; Hubbard, 1996; Klapetek et al., 2012; Marks, 1974, 1987, 1989), hue (e.g., Melara, 1989; Spence and Di Stefano, 2022a, for a review) and elevation (Bonetti and Costa, 2019; Wrembel, 2009; Chiou and Rich, 2012), etc. (see also Sun et al., 2018). The existence of such correspondences has often been explained in terms of the statistical or structural/physiological account (Spence, 2011).

There has, however, been widespread disagreement in the case of a putative crossmodal correspondence between auditory pitch and visual hue. Some commentators have been convinced that such a correspondence must exist based on the structural similarity of the underlying sensory dimensions (Gombrich, 1960; Sebba, 1991) while others deny the existence of such a correspondence, or else disagree concerning what the most appropriate mapping would be (Bernstein et al., 1971; Caivano, 1994; Davis, 1979; Garner, 1978; Newton, 1704). At the same time, however, other researchers have argued that methodological issues (i.e., confounding hue and brightness) may have compromised the interpretation of several earlier studies (O'Malley, 1957; Simpson et al., 1956; Wicker, 1968). Despite the structural features that might be shared by the visual dimension of hue and the auditory dimension of pitch (e.g., categorical perception in the continuous nature of wavelengths/frequency range scales), as highlighted in the reviews from Spence and Di Stefano (2022a, 2024a), there would appear to be little agreement as far as which hue should, in fact, be matched with which musical note. What is more, structural issues soon emerge when it comes to explaining color-sound correspondences based on any perceptual, psychophysical, or physical similarity between the two component stimuli. For instance, when considering the color spectrum and audible sound range, the former fundamentally differ in terms of the non-linear distribution of tones along the audible frequency spectrum as compared to colors in the visible range. Moreover, a fundamental property of auditory perception, namely octave periodicity, seems to have no parallel in vision, apart from some metaphorical and indirect usages of the same terms for referring to colors by some commentators (e.g., Pridmore, 1992).

More recent research has drawn attention to the existence of a number of robust crossmodal correspondences between visual features (i.e., surface textures) and auditory timbre, due to the allegedly intrinsic multisensoriality of timbre characterization/semantics (e.g., Reuter et al., 2018; Reymore and Lindsey, 2025; Saitis et al., 2020; Wallmark, 2019).^5^ For instance, Reymore et al. (2023) demonstrated that timbre semantic associations mostly refer to the visual appearance of objects, such as the material or haptic features of surface textures (e.g., raspy, smooth, brilliant). Several other recent studies have, though, deepened our understanding of the relationship between auditory roughness and shapes, by demonstrating that listeners tend to match more dissonant sounds to spikier and rougher objects/shapes (see also Murari et al., 2015). Giannos et al. (2021) assessed whether non-tonal and highly dissonant harmonic stimuli are associated with rough images, while more consonant stimuli are associated with the images of low visual roughness. To test this particular hypothesis, the researchers harmonized a fixed melody in seven different styles, including highly tonal, non-tonal, and random variations. The participants in this study were asked to match the melodies to images of variable roughness (i.e., black and white 2D and 3D images that represented surfaces with different degrees smoothness/roughness). In this case, the results confirmed that auditory dissonance was indeed highly correlated with visual roughness (see also Di Stefano and Spence, 2022, for a review).

Other researchers have demonstrated the existence of audiovisual crossmodal correspondences between musical timbre and visual shapes (Adeli et al., 2014; Gurman et al., 2021). For instance, the participants in a study by Adeli et al. (2014), were asked to select the visual equivalent of a given sound, i.e., its shape, color (or grayscale) and vertical position. A strong association between timbre and visual shapes was observed, with soft timbres being more frequently associated with blue, green or light gray rounded shapes, while harsh timbres were associated with red, yellow or dark gray sharp angular shapes. Importantly, these findings were subsequently replicated by Gurman et al. (2021).

### 2.2 Crossmodal effects

A wide range of crossmodal correspondence effects have been demonstrated across a wide range of experimental paradigms (e.g., Evans and Treisman, 2010; Gallace and Spence, 2006; Parise and Spence, 2012; and see Spence, 2011; Spence and Sathian, 2020, for reviews). So, for example, Parise and Spence (2009) published a series of psychophysical studies demonstrating increased spatiotemporal integration for crossmodally congruent combinations of auditory and visual stimuli (e.g., relative high-pitched sound with a relatively large visual stimulus) as compared to incongruent combinations (e.g., relatively high pitch and small visual size in an unspeeded psychophysical task).^6^ Sound frequency has also been reported to influence people's responses to color lightness (Hagtvedt and Brasel, 2016).

Other researchers have started to address the question of whether timbre modulates visual perception. So, for example, in a series of two experiments, Wallmark et al. (2021) investigated whether the timbre of a musical note (an acoustic prime) would affect the subsequent perception of, in the first experiment, brightness (dark–bright dimension) and, in a second study, both brightness and spatial texture (smooth–rough dimension). The participants had to identify a shift in roughness/brightness between two consecutively-presented target squares of subtly contrasting levels (rougher/brighter, smoother/darker, or the same) in a speeded-response paradigm. In a second experiment, prior to the presentation of the target square, the participants could be exposed to sounds that varied in terms of their roughness (smooth/rough). For visual stimuli, photos of sandpaper patches of contrasting grit sizes were used: baseline, medium roughness (100-grit); low roughness (150 grit); and high roughness (50 grit). A sine wave and a sawtooth wave they used as auditory stimuli. Modest evidence was found that timbres increase response bias in a semantically congruent manner when participants identify visual stimuli (e.g., when a “rough” saw-tooth wave accompanies the second of two identical spatial textures, the “rough” sound increased the probability of participants judging the second texture as rougher), thus suggesting that rough sounds may increase of the perceived roughness of the visual stimuli (see also Wallmark and Allen, 2020). Other researchers, meanwhile, have chosen to investigate timbral brightness by means of multisensory interference phenomena (Saitis and Wallmark, 2024).

Hence, in summary, there is currently robust experimental evidence for the existence of a wide range of audiovisual crossmodal correspondences involving pitch, such as pitch-size, pitch-brightness, pitch-height, with the exception of pitch-hue correspondences. In the latter case, despite the fact that many commentators are convinced of what the most appropriate crossmodal mapping is (see Spence and Di Stefano, 2024a, for a review), there would appear to be little agreement except in odd cases (such as the color scarlet supposedly matching the sound of the trumpet; e.g., Kandinsky, 1977 (originally published in 1911); Leibniz, 1896 (originally published 1704); Locke, 1690;^7^ Ortmann, 1933; see also Donnell-Kotrozo, 1978). However, the fact that pitch-based crossmodal correspondences are relative in nature for normal listeners (Spence, 2019),^8^ means the consequences for those wanting to art crossmodally are unclear, though the question remains open for those with absolute pitch (see Di Stefano and Spence, 2024b).^9^ What is more, the sheer range of crossmodal correspondences that might be primed by a given auditory pitch means that it is difficult to see how a unique mapping could be established in (e.g.,) a sensory-substitution setting (Cavazos Quero et al., 2021), or else when considering the question of sensory translation between one sense and another (Spence and Di Stefano, 2024b). This challenge presumably also applies for those wanting to translate works of art from one sensory modality into another (e.g., see Müller-Eberstein and van Noord, 2019).

## 3 Structural correspondences

Mid-level audiovisual crossmodal correspondences operate on dynamically-changing stimuli and/or on combinations of unisensory stimuli (attributes or dimensions; Guo et al., 2023). They sit between the correspondences that have been observed with individual stimuli and those that operate at the level of more aesthetically-meaningful complex sets of stimuli.^10^ Mid-level crossmodal matching is sometimes based on the common numerosity, or spatiotemporal organization, of the individual elements that happen to be presented in each sensory modality (see Spence, 2015, for a review), while at other times it may be based on correlated unisensory input (Parise et al., 2012, 2013). One might also think of crossmodal correspondence that are based on the harmony of the stimuli presented in each modality (Alves, 2005; Spence and Di Stefano, 2022b; Wells, 1980; cf. Pimentel et al., 2025). At the same time, however, metaphorical (e.g., Ravignani and Sonnweber, 2017; Wagner et al., 1981) and emotional mediation has also been reported in at least some cases.

### 3.1 Crossmodal matching

To date, far fewer crossmodal matching studies have been conducted at this level. What is more, the majority of research that has been published to date would appear to involve cross-level correspondences, such as music-to-color associations for single-line piano melodies (Lindborg and Friberg, 2015; Palmer et al., 2016). Researchers have assessed crossmodal correspondences between colors and music clips (Chen, 2013), musical genres (Holm et al., 2009; see also Bresin, 2005), music tempo (Guo and Jiang, 2023; see also Timmers, 2022), melody (Cuddy, 1985), and musical intervals Di Stefano et al. (submitted)^11^. Gaiger (2018) has even suggested that paintings can have rhythm. It is also easy to see how the emotional associations listeners have with major vs. minor chords could also be linked to colors (Parncutt, 2014, 2024; see also Carraturo et al., 2025; Wanke et al., 2025).

### 3.2 Crossmodal effects

When, for instance, the rate of repetitive auditory stimuli is increased or decreased, while a simultaneously-presented visual flicker remains constant, the latter appears to change accordingly with the auditory stimulus, an effect known as “auditory driving” (Gebhard and Mowbray, 1959; Recanzone, 2003, 2009; Shipley, 1964; Welch et al., 1986; see also Boltz, 2017, 2018; Occelli et al., 2011; Shams et al., 2000). By contrast, changes in the rate of visual flicker do not appear to change the perceived rate of auditory flutter to anything like the same extent. This, then, is an example of auditory dominance (see Di Stefano and Spence, 2025, for a number of other crossmodal influences in the perception of temporal structure).

O'Leary and Rhodes (1984) conducted a study demonstrating a bidirectional influence between vision and audition in a task involving perceptual organization. Multiple studies have demonstrated how intramodal perceptual grouping precedes crossmodal influences in the context of the crossmodal dynamic capture effect (Soto-Faraco et al., 2003; Spence et al., 2007; see also Cook and Van Valkenburg, 2009; Gilbert, 1938; Maeda et al., 2004; Takeshima and Gyoba, 2013; Watanabe and Shimojo, 2001).^12^ Crossmodal congruency effects have also been documented based on stimulus identity, in this case reflecting a short temporal pattern (Frings and Spence, 2010; cf. Huddleston et al., 2008; Julesz and Hirsh, 1972).

Consider here only how the affective meaning of musical scales depends on their direction—namely, whether they are ascending or descending (Collier and Hubbard, 2001). On the one hand, this association hints at the importance of the structural organization of the individual stimuli presented in a given modality, over-and-above whatever sensory qualities any of the individual elements may have when presented in isolation. This observation may also be linked to the relative nature of crossmodal correspondences, especially those involving auditory pitch (Spence, 2019; see also Eysenck, 1940). More broadly, the way in which individual elements are structured within a given sensory modality—and how they relate across the senses—brings us to the concept of harmony, a principle that has long been central to both music and visual aesthetics. In the Greek tradition, the term harmony originally related to the physical unification of different elements (Lippman, 1963). As such, the concept of harmony found a natural application in the domain of music, conceived of as an artistic practice based on the juxtaposition of different auditory elements (i.e., sounds) fitted together to generate pleasant effects. In the field of vision science, a number of researchers have investigated the concept of harmony between color pairs (e.g., Allen and Guilford, 1936; Burchett, 2002; Field, 1835; Palmer and Griscom, 2013; Schloss and Palmer, 2011). McNeill Whistler (1978) talked of paintings as harmonies (though see Kargon, 2011). Some suggest that music and painting may be combined harmoniously (e.g., consider only the literature on “color music”; Argüelles, 1972; Plummer, 1915; Rimington, 1915; Sullivan, 1914; Watkins, 2018; Zilczer, 1987). Given such considerations, it becomes natural to consider whether the concept of crossmodal harmony makes any sense (Kargon, 2011; see Spence and Di Stefano, 2022b, for a review).

## 4 Crossmodal correspondences between complex auditory and visual stimuli

Experimental psychologists have long been interested in the crossmodal matching of complex auditory and visual stimuli, as well as on the crossmodal effects of one on the other (see Spence and Di Stefano, 2025, for a review). For whatever reason, the majority of studies that have been published to date in this area would appear to have been conducted with images of paintings and short classical music selections (see Spence, 2020a, for a review).

### 4.1 Crossmodal matching

A number of studies have presented a small selection of stimuli in one sensory modality, with participants being required to match from a pre-selected set of stimuli presented in the other modality (see Table 3). It should, though, be noted that many of the early studies would appear to have been statistically underpowered by contemporary standards. Moreover, there have been relatively few attempts to replicate previous studies, as has become an increasingly common practice given the replication crisis in psychological science. That said, the majority of the research that has been published to date appears to demonstrate a consensual (i.e., a significantly non-random) tendency (though, of course, one should always be mindful of the so-called “file drawer problem”; see Rosenthal, 1979).

**Table 3:** Broad categorization of different types of audiovisual crossmodal matching and crossmodal effects involving complex multi-element aesthetically-meaningful sensory stimuli (see Spence and Di Stefano, 2025).

**Type of correspondence**	**Stimuli involved**	**Crossmodal matching (CM)**	**Crossmodal effect (CE)**
Complex (or high-level)	Paintings and pieces of music	Walker, 1927; Cowles, 1935; Wehner, 1966; Peretti, 1972; Minnigerode et al., 1976; Parrott, 1982; Albertazzi et al., 2015; Rančić and Marković, 2019; Iosifyan et al., 2022	Lindner and Hynan, 1987; Limbert and Polzella, 1998; Actis-Grosso et al., 2017; Rančić and Marković, 2019; Braun Janzen et al., 2023; Fekete et al., 2023; Fink et al., 2024

The majority of research that has been published to date would appear to be consistent with the claim that complex crossmodal associations between music and paintings are primarily mediated by emotion (see also Hung, 2000).^13^ More refined protocols typically present the auditory stimulus—specifically, a musical excerpt—while having the participants rate the extent to which it matched each of the pre-selected visual stimuli. This kind of experimental design allows researchers to better understand association trends (e.g., Di Stefano et al., 2024, 2025) while, at the same time, avoiding the risk of forcing a “best” choice response if none of the presented stimuli happen to clearly match the auditory excerpt. There have also been a number of studies looking at cross-level correspondences involving complex stimuli as but one component. For instance, Isbilen and Krumhansl (2016) conducted research highlighting the existence of emotion-mediated color associations to Bach's Well-Tempered Clavier. Furthermore, and similar to what has just been reported, the suggestion is that crossmodal correspondences between music and ambient color are mediated by emotion (Hauck et al., 2022; though see Lipscomb and Kim, 2004).

### 4.2 Crossmodal effects

Researchers have demonstrated that music (or musical emotion) can crossmodally influence the perceived (or at least rated) brightness of paintings (Bhattacharya and Lindsen, 2016; see also Logeswaran and Bhattacharya, 2009). For example, the participants in one study reported by Hong et al. (2024) were presented with a wide range of emotional music pieces alongside various visual stimuli. The results revealed that lower-pitched music tended to result in darker judgments of visual objects than when higher-pitched music was presented instead (again see Table 3).^14^ Meanwhile, a study by Albertazzi et al. (2020) investigated the existence of crossmodal correspondences between contemporary art (paintings by Kandinsky) and music (excerpts from Schönberg). The experiment was conducted in two phases. In the first phase, the participants evaluated the perceptual characteristics first of visual stimuli (some pictures of Kandinsky's paintings, with varying perceptual characteristics and contents) and then of auditory stimuli (musical excerpts taken from the repertoire of Schönberg's piano works) relative to 11 pairs of adjectives tested on a continuous bipolar scale (i.e., based on Osgood's semantic differential technique; Osgood et al., 1957). In the second phase, participants were required to associate pictures and musical excerpts. The results of the semantic differential demonstrated that certain paintings and musical excerpts were evaluated as semantically more similar, while others were evaluated as semantically more different. At the same time, the results of the direct association between musical excerpts and paintings showed both attractions and repulsions among the stimuli. The overall results provide significant insights into the relationship between concrete and abstract concepts and into the process of perceptual grouping in cross-modal phenomena (see also Ansani et al., 2020; Boswell, 2012; Ellis and Simons, 2005; Iwamiya, 1994).

### 4.3 Interim summary

Given the research that has been reviewed so far, it would appear clear that crossmodal matching occurs between all possible combinations of individual, organized, and more complex, semantically-meaningful stimuli. Having demonstrated such crossmodal correspondences the next question is how such findings inform attempts at sensory translation or sensory augmentation. At the outset here, one might consider whether crossmodal matching is perhaps more relevant when considering sensory translation, whereas the literature on crossmodal effects may be more pertinent for those wanting to augment art crossmodally. Returning to the two important claims that we put forward earlier, we might say that the mere existence of a crossmodal correspondence (as revealed by research on crossmodal matching), has no necessary implications for the existence of a crossmodal effect. A second claim is that the existence of crossmodal correspondences operating between individual sensory stimuli has no necessary implications as far as the existence of crossmodal effects operating between complex stimuli are concerned. Furthermore, it can also be argued that the absence of a demonstrable crossmodal effect cannot be taken as evidence of the absence of a crossmodal correspondence either. That is, it is at least theoretically plausible, that people might consensually rate certain combinations of stimuli as matching vs. mismatching, without there being any crossmodal effects on performance (e.g., in a speeded classification task). All three of these claims would appear consistent with the various sources of evidence that have been reviewed here.

## 5 Attempts to augment auditory/visual art and entertainment crossmodally

There is an extensive literature on “color music”, sometimes called “visual music” (e.g., Adams, 1995; Baker, 2002; Dimova, 2024; Galeyev, 2003; Klein, 1937; Liu, 2022; Lupton, 2018; Peacock, 1988; Poast, 2000; Sabaneev and Pring, 1929; Sabaneyev, 1911; Scholes, 1970; Shaw Miller, 2006; South, 2001; Vergo, 2012; Zika, 2013, 2018; Zilczer, 1987, 2016). These phrases refer to artistic interest in both translating music into color but also in augmenting one art form by additional stimulation in the other modality. When translation is attempted in the opposite direction it is sometimes referred to as “graphical sound” instead (Benčić, 2021). While it has been argued at length elsewhere that literal sensory translation may not be possible (Spence and Di Stefano, 2024a; see also Zika, 2013, 2018), there is an interesting body of artistic work that probes the possibility of augmenting art crossmodally (see Spence and Di Stefano, 2025).

Many of those interested in this area invoke the notion of synaesthesia when thinking about engaging multiple senses simultaneously (e.g., see Dimova, 2024; Karwoski et al., 1942; Spence and Di Stefano, 2025; Waterworth, 1997). However, as Deroy and Spence (2013) have previously argued at length, such an appeal (to synaesthesia) is fundamentally misguided, and that what may be much more important is to build on the crossmodal correspondences that are consensually shared by the majority of people if one wants to augment art crossmodally in a way that communicates meaningfully with the majority (cf. Spence, 2012). Polina Dimova uses the term “synaesthetic metaphor” as a heuristic device (Dimova, 2024), grouping congenital synaesthesia, literary synaesthesia (aka pseudo-synaesthesia), intermediality in the arts, and cultural synaesthesia together. As Howes (2025, p. 147) puts it, she: “engages with them contrapuntally, in the manner of a fugue. The right-thinking neuroscientist would be aghast at such heterodoxy and abominate it”. The reason being the Criterion of Consistency test that is now used as a gold standard to determine who is a “genuine synaesthete” according to neuroscience (Baron-Cohen et al., 1993; Johnson et al., 2013; Root et al., 2025; Svartdal and Iversen, 1989; though see also Harrison and Baron-Cohen, 1996). Such a view, although aligning with the “neuromania” (Legrenzi and Umiltà, 2011) or “cerebrocentrism” (Howes' terminology) of so much contemporary cognitive neuroscience research, can seem overly constraining when considering the crossmodal augmentation of art (e.g., see Howes and Classen, 2014; Young, 2005). What is needed is rather more of a social/structuralist account of synaesthesia, consistent with the views put forward by Lévi-Strauss (1997). Echoing much the same point, half a century earlier, Vanechkina (1973) noted how almost all of those he spoke to specially emphasized the associative, metaphoric nature of “color hearing” in music and excluded from the artistic sphere any clinical cases of color hearing.

Separate from any attempt to use synaesthesia in crossmodal compositions, it should be recognized that certain art forms, such as live opera, inherently stimulate multiple senses; Live music performance too, though this varies all the way from simple inevitability of seeing musicians play in a classic music concert through to the visuals that augment more experimental music (see also McDonald et al., 2022). In the latter cases, though, it is by no means always clear which is the dominant/primary modality. However, separate from these issues, there are genuinely unisensory experiences of art, painting, sculpture, at least as it is displayed in contemporary galleries.^15^ At the other extreme, there may be accidental/incidental background music presented in a gallery environment (see Table 4). In such cases, there is unlikely to be any intentional connection, or expectation of relevance or resonance (Loureiro et al., 2019).

**Table 4:** Summary of various kinds of audiovisual artistic/entertainment experience, where music complements/augments the visuals, or vice versa.

**Artistic/entertainment experience w. music**	**Relation between auditory and visual elements**	**Example (relevant literature)**
Attending a classical music concert in person	The seen gestures of the musicians and vocalists are the cause of the sounds that are heard	Vuoskowski et al., 2014, 2016
Dance/ballet performance, or a rhythmic gymnastics routine	Seen dancer's movements typically a response to the auditory score (e.g., The Nutcracker; or Swan Lake)	Chiat et al., 2013
Going to the opera	The background music is designed to set a mood for the on-stage action	Though see Kierkegaard, 1988, on Mozart's opera, Don Giovanni
Attending a performance of Scriabin's Poem of Fire with *luce* accompaniment	Music composed first and luce designed to enhance the experience	Scriabin, 1910; Gawboy and Townsend, 2012
Watching an audiovisual Film or TV advert	Music normally added post-production in order to convey a particular mood, emotion, or atmosphere	
Video-jockey (VJ); Jean-Michel Jarré concert (Boltz, 2013)	VJ mixes a visual accompaniment in response to pre-existing music	Synaesthetic visual augmentation of Mahler's 2nd symphony (Deutsch, 2012; cf. Haverkamp, 2020)
Multimedia artworks composed to engage both eye and ear	Audiovisual stimuli may be synchronized, and/or may share a common, or intentionally conflicting emotional message	Basquin, 2020; Benčić, 2021; Whitney, 1980
Experiencing architecture w. musical accompaniment	Given stylistic similarities, music sometimes used to augment videos of architecture	Muecke and Zach, 2007; Siefkes and Arielli, 2015
Visual artwork augmented by auditory (or other sensory elements)	The auditory augmentation is designed to modify the viewer's experience of the artwork; Some examples of “color music” or “visual music” fit here	Tate Sensorium (Pursey and Lomas, 2018)

Notice how the synchrony between the auditory and visual elements may sometimes exogenously encourage the integration of the audiovisual inputs (e.g., when attending a classical music concert; see also Muller, 2010), while in other cases, the context in which the two channels of communication are experienced may encourage more of an endogenous, or top-down, attempt by the viewer (or audience) to combine the multisensory inputs.

Iwamiya (2013) investigated the interaction between auditory and visual processing when listening to music in an audiovisual context. C. D. Friedrich, for example, exhibited pictures in low light accompanied by music (Siegel, 1974). P. O. Runge exhibited the *Times of the Day* painting series in J. W. von Goethe music room and by doing so, the suggestion is that the aesthetic experience was escalated into a full multisensory experience (The Getty Research Institute, 2013).

### 5.1 Sensory translation: turning music into light

There has long been artistic interest in the possibility of sensory translation (Kargon, 2011; see Spence and Di Stefano, 2022a, for a review). Relevant here, Stechow (1953) highlighted an important distinction between: “translations from the visual arts into music and parallelisms between the visual arts and music” (p. 324, italics in original). Later, he observed that “it would seem to me that comparability of structure reveals a more ‘real' relationship between such works of art than a mere affinity of ‘mood' or ‘texture' could suggest” (Stechow, 1953, p. 325); Notice how the latter comment presumably emphasizes the structural rather than the affective nature of underlying correspondences (though see Kargon, 2011, on the contrary position). Just consider Alexander B. Rimington (1854–1918) of the Royal College of Arts in London, who first presented his color organ to the public in 1895, and which he performed with up until 1911 in London (Rimington, 1895, 1911, 1915). Father Castel, the French priest's earlier suggestion that every home would one day have an ocular harpsicord never came to pass (see Conrad, 1999; Moritz, 1997). The project was likely doomed to failure (Hankins, 1994).^16^ That is, the best that you can hope to do is to convey the emotional tone of a specific work (again, see Spence and Di Stefano, 2022a, 2024a, for reviews). Furthermore, sensory translation has typically been considered in terms of the element-by-element translation of individual sensory stimuli (Gibson, 2023; Spence and Di Stefano, 2024a). However, as we have just seen, mid-level and complex correspondences emerge as a result of intramodal perceptual grouping, and hence what might be more appropriate to translate is the unimodal Gestalt, in the augmentation of art and entertainment crossmodally (Bragdon, 1916, 1918).

### 5.2 Sensory augmentation: Scriabin's Poem of Fire

A somewhat distinct literature has emerged on the question of the crossmodal sensory augmentation of art. Here, one might consider Scriabin's (1910) “Prometheus: Poem of Fire” as a paradigmatic example (Hull, 1927). This musical score is unusual in that it came with an accompanying score for the *luce* (Brent-Smith, 1926a,b; Gawboy and Townsend, 2012).^17^ Although not performed in Scriabin's lifetime, the code for the light score (which had been thought lost) was discovered some decades later (Galeyev and Vanechkina, 2001). Unfortunately, however, Scriabin died before the first performance of his work with *luce* accompaniment took place (in New York City in 1915; Baker, 2002). Nevertheless contemporary performances of this multisensory artwork have sometimes incorporated both auditory and visual elements (Galeyev, 1988; see Spence and Di Stefano, 2022a, for a review). One can think of this as an attempt to augment (i.e., rather than to translate) music with a color organ. That said, Scriabin's intention for the *luce* remains shrouded in mystery. Confusing matters somewhat, Scriabin himself was a synaesthete (including color music; Triarhou, 2016; Witztum and Lerner, 2016).^18^ Some commentators have suggested that the introduction of the *luce* may have been intended to help disambiguate elements in the musical score (Gawboy and Townsend, 2012). However, this remains a question of ongoing theoretical debate (see also Howes, 2025).

Intriguingly, although formal evaluation has not yet been conducted, those commentators who have experienced such intentional multisensory, or crossmodal, artistic presentations have, on occasion, hinted at an emergent experience, one that supports the role of Gestalt perceptual grouping in a crossmodal artistic context (though note that the rich notion of multisensory emergence likely reaches beyond the insights offered by the Gestalt approach). Just take the following quote from Wells (1980, p. 101–105): “One has only to trace the remarkable unfolding of harmonic color in Scriabin's later works to realize that it was harmony above all that prompted him to seek a visual counterpart to his music. An equivalence of aural harmony and visual is so strongly implied in Scriabin's later works that for me they seem to emanate from a single source…The author tells of events leading up to finding a possible link between musical harmony and visual color based on complementarity…The parallelism of musical harmony with visual color departs radically from current hypotheses of tonal organization.” (cf. Spence and Di Stefano (submitted)^19^, on the notion of crossmodal counterpoint). Note here, though, that Wells is not a disinterested observer inasmuch as in his article he goes on to propose a crossmodal pitch-hue mapping of his own.

In principle, multisensory emergence might be just one example of an extraordinary multisensory experience (e.g., Critchley, 1977; Ehrenzweig, 1965). Sirius and Clarke (1994, p. 119) similarly write of how: “There is a long history of the association of music with visual spectacles of one sort or another (opera, theater, and in this century film, television and video), and a substantial amount of anecdotal evidence that the two media can interact in powerful and effective ways.” (see also Anderson, 1993). The unification of the unimodal elements (as hinted at by Wells, 1980) is another possible outcome that is sometimes mentioned in the literature albeit infrequently, as is the notion of resonance (perhaps a kind of crossmodal modulation) is also mentioned in more artistic contexts (e.g., Muecke and Zach, 2007; cf. McQuarrie and Mick, 1992). Researchers have demonstrated that visual cues can modulate the integration and segregation of objects in auditory scene analysis (Rahne et al., 2007). Audiovisual Gestalten likely rely as much on temporal synchrony as much as any crossmodal correspondence at the level of individual sensory elements, or even the Gestalt impression (see also Alves, 2005; Battey and Fischman, 2016; HaCohen, 2016; Haverkamp, 2020; Kaduri, 2016; Muller, 2010; Russet and Starr, 1988; Whitney, 1980). At the same time, however, it has also become increasingly clear that there is no straightforward structural or psychological mapping between audition and vision.

While the term multimedia is broad, and covers multiple forms, there is a sense that some multimedia art works fit the description of crossmodal art in that the stimuli presented to eye and ear are in some sense independent, yet the case is different from the addition of music to film, where the music is seemingly always added as an afterthought (Lipscomb, 2013; Tan et al., 2013; Truckenbrod, 1992). At the same time, however, there are empirical phenomena that are undoubtedly worthy of some form of explanation. Consider here only the phenomenal success of the music of Jean-Michel Jarré, with the largest live audience for an entertainment event ever recorded at the time (in excess of 750,000 people; cf. Boltz, 2013; Tan et al., 2013) when a light show would accompany his musical performances. It would seem hard to explain why such multimedia performances were (at least for a time) so successful, without having recourse to some kind of emergent experience that elevated the multisensory experience beyond what would have been experienced at a traditional music concert (or color organ show; see Spence and Di Stefano, 2022a).^20^ At the time, these were the largest live entertainment audiences ever recorded, and thus speak to the power of simultaneous audiovisual stimulation.^21^ In this case, the artist was augmenting his music, which came first, with visuals (see also Collopy, 2000, and Chmiel and Schubert, 2019, for a history).

### 5.3 Intersensoriality

Howes (2025) has recently introduced the notion of intersensoriality to describe art forms that deliberately cross sensory borders (Classen, 1998). The notion of intersensory, somewhat distinct from both multisensory integration and synaesthetic translation, emphasizes relationality, complementarity, and emergent meaning. Here, the senses are not unified in an essentialist manner, nor merely co-activated, but brought into dialogue in a way that preserves and indeed accentuates their difference. Howes (2025, p. 143) writes of how: ““the new intersensory music and art history” departs from the conventional distribution and hierarchization of the sensible by focusing on the exchanges between—and intercalibration of—the senses. This redirection of attention is manifest in such works as Classen's (1998) chapter on “Crossing sensory borders in the arts” in The Color of Angels, Shaw-Miller's (2010) Eye hEar: The Visual in Music, and Veldhorst's (2018) Van Gogh and Music: A Symphony in Blue and Yellow, among other works (see especially Lauwrens, 2012).” Howes (2025, p. 147) concludes his piece as follows: “here was an exhibition entitled “The Romantic Eye” going on at the Swedish Nationalmuseum at the time of the Stockholm conference. It was a little too ocularcentric for my taste, but one writing on a wall caught my eye: “‘An artist is each and every one for whom the aim of life is to develop one's senses'—Friedrich Schlegel”. Fair enough, but for the avant-garde it's different: for them, making music or making art involves straining at the bounds of sense, or crossing over (Classen, 1998). Contemporary historians of music and art need to follow suit if they are to make any headway in the understanding of the art-music nexus at the conjuncture of the nineteenth and twentieth centuries as at the conjuncture of the twentieth and twenty-first centuries (see Jones et al., 2006; Lauwrens, 2012). As we have seen, it is the renvoi, the interplay, the interrelationships of the senses that define the most lasting aesthetic expressions of the two periods”.

The notion of intersensoriality can thus help to promote a relational view of the senses, one that resonates with Lévi-Strauss' (1997) idea, expressed in *Look, Listen, Read*, that systems of perception in different senses can be homologous without necessarily being identical. In particular, his commentary on Father Castel's color organ (see Lévi-Strauss, 1997, p. 129 and ff) exemplifies this: the device did not conflate sound and color into a single unified percept, but rather highlighted their relational differences. This relational interpretation also aligns with Baumgarten (1750) interpretation of aesthetics as, in Leibnizian terms, the capacity to grasp “unity-in-multiplicity” among sensible qualities. Here, one might also consider Septimus Piesse's “Gamut of Odors” (Piesse, 1867) in which he matched 24 musical notes to a range of scents. While a casual reading might suggest that Piesse is proposing a direct crossmodal mapping between specific musical notes and odors, a closer reading of this English chemist's work, suggests instead that he had more of a relational mapping in mind; meaning that successful combinations of odors can be predicted on the basis of pairing associated notes that go well together. In his treatise, Piesse explicitly noted how sounds and odors blend together similarly, producing different degrees of “a nearly similar impression” in the sensory nerves (Piesse, 1867, p. 39). Piesse also writes about how the mixture needed to prepare the odors for the handkerchief evokes effects on the smelling nerve “similar to that which music or the mixture of harmonious sounds produces upon the nerve of hearing, that of pleasure” (Piesse, 1867, p. 219). He also claims that creating a mixture of scents is like creating a mixture of sounds, i.e., chords. At one point, he even writes that: “We have citron, lemon, orange peel, and verbena, forming a higher octave of smells, which blend in a similar manner” (Piesse, 1867, p. 39). Piesse was seemingly convinced that the pleasantness of musical harmony resembles that of perfumes (consisting of various base notes), and he presented a scale of correspondences between sounds and odors, as he believed that “there is, as it were, an octave of odors like an octave in music” (Piesse, 1867, p. 38).

In this light, mid-level crossmodal correspondences deserve renewed attention—not as mere by-products of low-level sensory matching or high-level conceptual metaphor, but as hybrid, emergent forms of intersensory pattern recognition. One key research question here thus becomes: What sensory feature, structural property, or dynamic pattern drives these mid-level correspondences? They often resist easy categorization because they operate at the cusp of the perceptual and the conceptual, exhibiting characteristics of both. One might refer here to the “collideroscopic sensorium” (Howes, 2023) as a useful metaphor for understanding how such intersensory correspondences open novel aesthetic dimensions without collapsing sensory boundaries.

### 5.4 Augmenting cinematic entertainment

In their 1928 “Statement on Sound”, Eisenstein, Pudovkin, and Alexandrov advocated the role of sound in film and for “the creation of a new orchestral counterpoint of sight-images and sound-images” (see Eisenstein et al., 1999). Still more radically, in an essay called “Synchronization of Senses”, Eisenstein describes how a “single, unifying sound-picture image” might be developed as a “polyphonic structure” that “achieves its total effect through the composite sensation of all the pieces as a whole” [Eisenstein, 1947; see Spence and Di Stefano (submitted)^19^]. Pointing to examples of synesthetic art including Rimbaud”s “color sonnet” and James NcNeill Whistler”s “color symphonies”, Eisenstein proposed that such effects could also be achieved in the cinema. At one point Eisenstein writes that: “To remove the barriers between sight and sound, between the seen world and the heard world … To bring about a unity and a harmonious relationship between these two opposite spheres. What an absorbing task! The Greeks and Diderot, Wagner and Scriabin-who has not dreamt of this ideal?” It is the ideal of the Gesamtkunstwerk, the total work of art that synthesizes multiple media: precisely the reverse of the Laocoön argument (see also Cardullo, 2017; Kandinsky, 1977; Smith, 2007). Here, note we see common themes emerging across different artistic/entertainment media.

Note that film and advertising are different inasmuch as people assume that the music and visuals have been deliberately paired in order to achieve some emotional effect or convey some form of semantic meaning (see Bolivar et al., 1994; Boltz, 2001, 2004; Boltz et al., 1991; Bullerjahn and Güldenring, 1994; Chion, 1990, 2000; Clemente et al., 2023; Cohen, 1990, 2010; Herget and Albrecht, 2022; Hung, 2000; Klein et al., 2021; Lipscomb and Kendall, 1994; Lipscomb and Tolchinsky, 2005; Millet et al., 2021; Nosal et al., 2016; Parke et al., 2007; Silas et al., 2024; Steffens, 2020; Thayer and Levenson, 1983; Tan et al., 2017, 2007; see also Carr and Rickard, 2016; Marin et al., 2012). For instance, Hung highlights how music in TV advertising can be analyzed in terms of its sensual properties, but also in terms of its emotional effect, and there again at the level of any semantic effects.^22^ Meanwhile, Gorbman (1987) suggested that any music applied to a film segment will affect the spectator because the latter automatically imposes meaning on such combinations. As such, there is likely to be an important difference between (artistic) media. Nevertheless, it is still interesting to note how many of the same themes and experimental manipulations (e.g., relating to the impact of congruent vs. incongruent combinations of film content and music stimuli) crop up in the literature on the effect of music on visual perception. Unfortunately, however, there is simply no opportunity (nor space) to review this parallel literature here. All we can do here is to highlight the existence of this large and highly-relevant experimental literature.

## 6 Challenges to the crossmodal augmentation of art

Before concluding this narrative historical review, it is worth noting how there are a number of challenges, theoretical, practical, attentional, and potentially also ethical when it comes to the crossmodal augmentation of art.

### 6.1 Theoretical challenges

A clear distinction between crossmodal augmentation in art (as in Scriabin, 1910, *Poem of Fire)* and apparently related attempts to translate auditory stimuli into visual ones, as in the case of Rimington's Color Organ (see also Kargon, 2011), should be highlighted here. In the latter case, at least according to the creator's intention, we are dealing with the straightforward attempt as sensory translation between audition and vision (Spence and Di Stefano, 2024b). In the case of Scriabin, there is a possible translation based on the composer's own color-music synaesthesia followed by a combination of the two stimuli, possibly in order to help disambiguate certain elements of the musical composition visually. It is, however, likely that some attempts at augmentation may result in nothing more that redundancy of information across sensory domains—similar to both seeing a battle and reading a description of it. This is what Banes (2001) describes, in the case of olfactory stimuli being used to provide the smell of what can be seen on stage, as pleonastic. It can be argued, by contrast, that crossmodal augmentation should by definition involve, more than mere translation or redundancy; it requires the addition of non-redundant sensory inputs from a different domain, enriching (or possibly disambiguating) the original experience.

Relatedly, one might draw attention to issues related to the sensory nature of artworks by observing that to have either sensory translation or crossmodally augmented art, the mere simultaneous perception of two sensory inputs is insufficient. For example, hearing Beethoven while viewing a Munch exhibit does not constitute sensory translation or augmentation. And what if a contemporary artist *deliberately* broadcasts Beethoven during the exhibition, explicitly presenting it as multisensory art, a ticket paid for it, and people made aware of this? Would that turn the mere coexistence of music and paintings into an example of crossmodal art? Let us return, then, to Danto (1981) with his key questions on the ontology of art, such as, for example: What makes something an artwork? What distinguishes art from mere objects? How does context shape art's identity? Danto's reflections challenge the idea that art is defined by its material properties alone, emphasizing instead its conceptual and contextual dimensions.

The crossmodal augmentation of art can therefore be seen as requiring more than mere than the simultaneous presentation of the relevant auditory and visual stimuli. It involves a deliberate, non-redundant integration of both, framed within an artistic context that invites interpretation. Crucially, the observer's awareness of this intentional combination transforms the experience from mere coexistence into a meaningful, augmented artistic phenomenon.

### 6.2 Practical challenges

It is worth pausing here to consider Behne's (1999) “habituation hypothesis”, according to which the effect of background music has steadily decreased due to the growing omnipresence and availability of music in day to-day life. In a meta-analysis, Behne observed that the effect of music on emotions, perceptions, and behavior had decreased by roughly 8% per decade. Such habituation, were it to be a replicable phenomenon, would obviously raise a practical challenge for those wanting to augment visual art with music, say. That said, Kämpfe et al. (2011) were unable to replicate this general historical trend in a more recent and more methodically controlled meta-analysis.

Separately, should any putative crossmodal augmentation rely on precise crossmodal temporal synchronization then there may be challenges around ensuring that different sensory inputs/elements align perfectly in real time (e.g., matching sound with visual effects). This can be problematic: Interactive or AI-driven experiences sometimes suffer from technology-induced lag in one or more senses, thus reducing the immersiveness of the experience. One might be reminded here of Bruce Naumann's (1969) artwork “Lip Sync” (see https://www.moma.org/collection/works/107669). The work consists of a close-up of a male face presented upside down repeatedly say “lip-sync”. The key point is that the asynchrony between the sound of the voice and the associated lip-movements continuously change in terms of the asynchrony thus drawing the viewer is as they can never quite pin down the relationship between the auditory and visual channels. One might consider this a genuinely audiovisual work of art, and as such, the notion of crossmodal augmentation doesn't really have any relevance.

### 6.3 Attentional constraints and the danger of sensory overload

The Danish philosopher Kierkegaard (1988, originally published in 1843) once wrote that: “It is a common experience that to strain two senses at the same time is not pleasant, and thus it is often disruptive to have to use the eyes a great deal at the same time as the ears are being used. Therefore, one is inclined to shut the eyes when listening to music. This is more or less true of all music, and *in sensu eminentiori* [in an eminent sense] of [Mozart's opera] Don Giovanni. As soon as the eyes are involved, the impression is disrupted, for the dramatic unity that presents itself to the eye is altogether subordinate and deficient in comparison with the musical unity that is heard simultaneously. My own experience has convinced me of this”. One might think of a view of artistic experience as grounded in sensory fragmentation compared to the multisensory inputs we ordinarily have to manage in daily life.

One argument suggested here is that part of what makes art powerful is its ability to isolate, emphasize, or distill specific aspects of perception. Unlike daily life, where we are bombarded with multisensory input that we must constantly filter, artistic experiences often provide a kind of structured reduction—a selective framing of reality that allows for deeper contemplation. For instance, a still life with fruits and bottles strips away movement, sound, odors, and so forces us to focus on composition, color, and form instead. This could explain why we value these aesthetic experiences, because they allow us to maximize sensory engagement. Reintegrating sensory modalities in aesthetic experience to augment them implies adding more sensory information (haptics, olfaction, spatial audio, interactive visuals, etc.). The danger is that this may merely increase cognitive load, making it harder to focus on any one aspect, thus undermining the essence of art and artistic contemplation. Is multisensoriality in art a form of redundancy—where different sensory modalities repeat the same message—or does it truly open new aesthetic dimensions?^23^

The argument seemingly has some cognitive implications. Freeing the senses from the burden of managing excessive stimuli implies freeing intellectual resources for deeper reflection and cognitive engagement. A key argument in favor of art as sensory fragmentation is that it does that to free the mind, allowing for more profound conceptual engagement. By narrowing the range of sensory input, art can liberate cognitive capacity for interpretation, imagination, and emotional resonance. In minimalist art, the deliberate reduction of visual complexity forces the viewer into an active intellectual engagement with form and space. By contrast, technologically-augmented multisensory experiences might do the opposite. Consider only the use of virtual/augmented reality (VR/AR) to augment aesthetic experience (e.g., Cho et al., 2020; He et al., 2018). If the mind is busy processing an excess of sensory inputs, does it have the capacity left to engage deeply with the ideas behind the work? One conclusion that could be drawn here is that while more sensory input might feel like an enrichment, it can also lead to passivity if the experience becomes too immersive or overwhelming. Consider here only Aitamurto et al.'s (2018) study in which the impact of a mobile video see-through AR tour guide on users' engagement with art was assessed. Besides showing many positives related to the AR technology itself, the results highlighted the downsides of the same technological augmentation related to the users feeling distracted by the AR guide and worried about excessive screen time.

Certainly, not all theorists/critics have necessarily appreciated the desire to engage more senses in art: As Arnheim stated in an essay entitled, with clear reference to the distinction among the arts in Lessing's 18th-century aesthetics, A New Laocoön: “in their attempts to attract the audience, two media are fighting each other instead of capturing it by a united effort” (Arnheim, 1957, p. 199; Babbitt, 1910)—this sounds like a problem of divided attention, and indeed problems of working memory have been suggested as a limiting factor when auditory and visual inputs are somehow independent. So, while in the best-case scenario, some sort of multisensory emergence may be experienced in the context of crossmodal art, there is always the danger of sensory overload should the various unisensory inputs not be sufficiently congruent (Malhotra, 1984). One other practical challenge that it is worth noting relates to the observation that deliberate attempts to enhance experience by deliberately introducing additional sensory inputs (e.g., in the field of sensory marketing) have, counterintuitively, often proved unsuccessful (Spence, 2021).

### 6.4 Is it ethical to deliberately augment a work of art crossmodally?

Finally, it is worth briefly considering the ethical question that was raised by David Lomas (from the Art History department at the University of Manchester), in relation to Flying Object's crossmodal augmentation of four works of visual art from the permanent collection at Tate Britain through the use of scent, sound, virtual touch, and even chocolate in 2015 (see Pursey and Lomas, 2018). Given that the artists of the original works were all dead by the time of the exhibition, one can ask the question of whether it is ethical to deliberately try to modify the meaning/viewer's experience of these important works of art by deliberately augmenting then through some form of crossmodal stimulation? Nevertheless, the situation feels importantly different from those cases where the original artist chooses to augment their own work (think Scriabin, and Jean-Michel Jarré). At the same time, the augmentation is clearly differentiated from the original experience. Here, one might consider whether one arrives at a different conclusion when thinking about the crossmodally augmented art as a (partially) different artwork. Consider here Eugène Bataille's, *La Joconde fumant la pipe*, (1897) or Duchamp's ready-made depicting Leonardo's Mona Lisa with mustache (*L.H.O.O.Q, 1919*) or Dalì's later alteration (Self-portrait, 1954).

## 7 Conclusions

One of the key points to emerge from the present narrative historical review is that it may not be possible to use elemental correspondences (that is, crossmodal correspondences based on simple stimulus properties), given that works of art are mostly complex, multi-elemental, and semantically/emotionally meaningful. This conclusion seemingly contradicting Eisenstein's suggestion that there is no “pervading law of absolute meanings and correspondences between colors and sound.” (as quoted in Harrison, 2001, p. 133–134). One might also want to consider whether crossmodal effects are more likely to be experienced if the auditory and visual stimuli are clearly linked (i.e., rather than, say, when music is treated as no more than incidental background music; see Liu, 2022). Returning to the main claims that were made in the Introduction: The division into basic, mid-level, and complex crossmodal correspondences certainly appears helpful when considering crossmodal correspondences/effects, especially in the context of translation/augmenting art experiences. At the same time, our sense is that more impressive outcomes are likely to be achieved should researchers and practitioners move away from the (idiosyncratic) synaesthesia mind-set, and rather focus instead on the consensually share crossmodal correspondences (see also Kargon, 2011). It will undoubtedly be interesting to see how this “intersensoriality” in art develops now that theorizing has moved away from the distraction of synaesthesia that hindered the first wave of interest a little over a century ago (Dimova, 2024; Howes, 2023, 2025).

One question for the future that we haven't had the opportunity to consider here is whether musical knowledge/expertise may affect audiovisual crossmodal correspondences and hence the way in which music and visuals interact in experience (Di Stefano et al., 2024, 2025; Di Stefano et al. (submitted)^11^; Guo et al., 2023; Mok, 2022; Vanechkina, 1968; Walker, 1987; see also Cutietta and Haggerty, 1987). Ultimately, though, as we have just seen, there are a number of theoretical, practical, attentional, and ethical questions that may sometimes arise for those interested in augmenting works of auditory or visual art crossmodally.

## Footnotes

^1^As Stein et al. (1996) note in one of their articles: “there is no animal in which there is known to be a complete segregation of sensory processing”.

^2^Note that while the descriptor “structural” might appear appropriate for many mid-level correspondences, the term “structural correspondences” has already been used in several other contexts previously (see Bernard, 1986; Sebba, 1991; Spence, 2011). Hence, to avoid confusion, we will use the descriptor mid-level instead.

^3^Here supporting the early position adopted by Helmholtz (1878/1971, p. 77), and *contra* Marks (2011; cf. Hartshorne, 1934). In brief, Helmholtz claimed that sensory modalities are fundamentally distinct, preventing direct transitions or comparisons between them. For instance, one cannot determine whether sweetness is more similar to red or blue. Comparisons are meaningful only within the same modality, such as identifying yellow as closer to orange than to blue.

^4^It is worth noting that, in similar protocols, the existence of a statistically significant crossmodal correspondence between stimuli in two arbitrary perceptual dimensions (such as pitch and hue; Walker-Andrews, 1994) only demonstrates that the chosen pairing was the best of the options that happened to be available to participants at the time they were asked (see Spence and Levitan, 2021).

^5^And see Menouti et al. (2015) for the synesthetic angle to this phenomenon.

^6^That said, Sourav et al. (2025) were unable to replicate at least one of these specific findings.

^7^Note though that Locke does not present this example in order to endorse its solution, but to comment ironically: “For the hope to produce an *idea* of light or color by a sound, however formed, is to expect that sounds should be visible or colors audible, and to make the ears do the office of all the other senses. Which is all one as to say that we might taste, smell, and see by the ears: a sort of philosophy worthy only of *Sancho Pancha*, who had the faculty to see *Dulcinea* by hearsay.” (Locke, 1690, pp. 29–30—emphasis in the original).

^8^Note, though, that the relative nature of crossmodal correspondences might not be restricted to pitch (see Cohen, 1934).

^9^Note though that the Pantone color of the year a few years ago was released along with a matching soundscape.

^10^One challenging case here is consonant and dissonant stimuli (Di Stefano et al., 2022). Some consider dissonant stimuli unitary, others believe that people are aware of the component stimuli.

^11^Di Stefano, N., Ansani, A., Schiavio, A., Saarikallio, S., Toiviainen, P., Brattico, E., et al. (submitted). “Minor seconds are bitter and blueish while major sixths are sweet and yellowish: a cross-cultural study of multisensory mappings of musical intervals,” in *Cognition*.

^12^Of course, it is entirely possible that any Gestalt perceptual grouping might only occur unimodally in the auditory and/or visual modalities, and crossmodal art might emerge from the parallel unisensory Gestalt perceptual grouping observed in each of the component modalities (cf. Kubovy and Yu, 2012)—one might consider this a kind of crossmodal correspondence between unisensory Gestalt attributes (see also Bender et al., 1954; Spence et al., 2007).

^13^That said, spatial conceptual associations between music and pictures have been revealed by researchers looking at the N400 effect (Zhou et al., 2014).

^14^It might be worth considering whether the concept of halo dumping (Clark and Lawless, 1994) could be relevant in those studies where the participants are only given a single dimension on which to respond.

^15^Though it is perhaps worth noting here that some early sculptural pieces were explicitly designed to be felt not seen (see Gallace and Spence, 2014). The shiny patina on many public brass sculptures clearly speaks to people's desire to haptically interact with many works of art when allowed.

^16^As was the *Pyrophone* from Kastner (1870) that incorporated 13 foil-covered gas nozzles, lighted by crystal tubes (Kastner, 1875).

^17^The piece was initially conceptualized as a total work of art combining music, a light score or “luce”, and even fragrance (Dimova, 2024; Peacock, 1985; Plummer, 1915; Scriabin, 1910). Although what fragrance Scriabin intended to use was never specified by the composer.

^18^In fact, he was tested by C. S. Myers at the University of Cambridge, and the results written-up in a couple of academic papers (Myers, 1911, 1914).

^19^Spence, C., and Di Stefano, N. (submitted). Crossmodal counterpoint: From music to multimedia – Incongruency, cognitive dissonance, irony, and surrealism. *i-Perception*.

^20^I.e., just as has been suggested in the case of Scriabin due to the crossmodal correspondence between light and sound in Poem of Fire (Wells, 1980; see also Boltz, 2013; see also Spence and Di Stefano, 2025).

^21^Although such audiovisual entertainments seemingly lost some of their magic subsequently.

^22^Note that semantic correspondences have also been shown to influence perception crossmodally (see Chen and Spence, 2010, 2011).

^23^One might also be reminded here of Nordau's (1898) suggestion that synaesthetic art (as popularized in the contemporary context by the likes of Waterworth, 1997; Whitelaw, 2008) was somehow degenerate (Nordau, 1898).
